# Surgical management of insulinomas at the Azerbaijan Medical University: a retrospective study of 21 cases over a 10-year period

**DOI:** 10.3906/sag-2001-150

**Published:** 2020-08-26

**Authors:** Rovshan HASANOV, Elgun SAMADOV, Nuru BAYRAMOV, Aytekin ÜNLÜ, Patrizio PETRONE

**Affiliations:** 1 Endocrinology Clinic, Leyla Medical Center; Baku Azerbaijan; 2 General Surgery Clinic, Leyla Medical Center; Baku Azerbaijan; 3 General Surgery Department, Azerbaijan Medical University; Baku Azerbaijan; 4 Department of General Surgery, University of Medical Sciences, Gulhane Medical University; Ankara Turkey; 5 Department of Surgery, NYU Langone Health – NYU Winthrop Hospital; NYU Long Island School of Medicine; Mineola, NY USA

**Keywords:** Pancreas, insulinoma, diagnosis, surgical treatment

## Abstract

**Background/aim:**

This study represents the first report that evaluates the experience gathered from diagnosis, surgical treatment and outcome of insulinoma patients from Azerbaijan.

**Materials and methods:**

We retrospectively review of insulinoma patients for a 10-year period. Collected data included patient demographics, laboratory and imaging tests, detailed surgical reports, histopathological examination of resected specimens, and clinical follow-up.

**Results:**

Twenty-one insulinoma patients were identified. Male patients comprised 52.4%; mean age was 44 years. Mean time to diagnosis was 14 months; 61% patients had ≥3 medical referrals due to hypoglycemia-related symptoms. Diagnosis sensitivity of CT, MRI and US was 85%, 80%, and 55%, respectively. The mean glucose, insulin, C-peptide levels were 35.7 ± 9.5 mg/dL, 33.5 ± 21.9 µU/mL, and 3.74 ± 1.88 ng/mL, respectively. Pancreatic head and tail were the most frequent tumor locations; mean tumor size was 1.5 ± 0.7 cm. No statistical association was found between the tumor size and preoperative glucose, C-peptide and insulin levels. Distal pancreatectomy and enucleation were the most common surgical procedures. Local tumor recurrence rate was 14%. There was no mortality.

**Conclusions:**

To prevent delayed diagnoses, physicians should be familiar with the typical symptoms of these rare tumors.

## 1. Introduction

Insulinomas are rare pancreatic neuroendocrine tumors (PanNET), which are found in 1–4 per million people each year [1,2]. Although PanNet account for less than 3% of all pancreatic tumors, their incidence had increased in the past two decades due to the advances in tumor localization capabilities [3,4]. PanNET can be functional or nonfunctional tumors depending on the presence of symptoms associated with hormone overproduction [5]. The most common functional PanNET is characterized by excessive amounts of insulin secreted [6]. Classically, these patients present with the Whipple’s triad, consisted of (a) hypoglycemic symptoms, (b) hypoglycemia (blood glucose level <50 mg/dL), and (c) relief of symptoms following administration of glucose. Patients may also present with muscle cramps, neuroglycopenic symptoms such as syncope, confusion, dizziness, and adrenergic symptoms such as tremor and tachycardia. Despite the classical symptoms, diagnosis of these tumors is often delayed in clinical practice. Surgical treatment strategies involve enucleation and resection of the tumor, although percutaneous ablation of lesions not amenable to surgery or chemotherapy for metastatic disease are also included [6,7]. 

Report of case series are mostly retrospective in nature and critical for rapidly developing and transitioning countries such as Azerbaijan for future comparisons. The aim of this study is to report the experience on PanNET at Azerbaijan Medical University.

## 2. Material and methods

This is a retrospective case series study approved by the Azerbaijan Medical University Ethical Committee (6 September 2018/C0234). Patients who underwent surgery at the Azerbaijan Medical University between March 2007 and April 2017 and had histopathological diagnosis of insulinoma were included in the study. Medical records of these patients were reviewed for data that include demographics, elapsed time between symptoms onset and diagnoses, location and size of insulinomas, diagnostic imaging methods, surgical reports, histopathological examination reports of resected specimens, postoperative complications for descriptive purposes. 

Data showed that all patients had a 72-h fast test for diagnosing insulinoma. During the test, upon finding a plasma glucose level of less than 45 mg/dL and hypoglycemia symptoms and findings, blood samples for plasma glucose, insulin and C-peptid levels were taken. The biochemical diagnosis of insulinoma was established during prolonged fasting (up to 72 h) when the following results were found: insulin level >3.0 µU/mL (18 pmol/L), C-peptid level >0.6 ng/mL (0.2 nmol/L), and plasma glucose level <45 mg/dL.

The abovementioned tests were followed by imaging tests to visualize the pancreas. Localization tests that include transabdominal ultrasound (US), computed tomography (CT), magnetic resonance imaging (MRI), arterial calcium stimulation and venous sampling (ASVS) and In-111 octreotide scintigraphy were also documented [8]. The palpation of whole pancreatic gland was also deemed helpful by the authors of this study, which occasionally was accompanied by intraoperative ultrasonography upon suspicious findings.

The choice of surgery depended on the location and size of the tumor. Superficial and encapsulated tumors were enucleated. Pancreatectomy was performed in cases with neck and proximal body insulinomas situated deep in the pancreatic gland and/or tumor location in close proximity to the main pancreatic duct. Extent and type of the resection was up to the surgeons’ choice. Pancreatic fistulas were classified according to the Study Group on Pancreatic Fistula grading [9].

Pathological examination of specimens was performed using conventional histologic and immunohistochemical (chromogranin A and synaptophysin) techniques. Ki67 index was expressed as a percentage of Ki67-positive cells in 2000 tumor cells.

Follow-up data was gathered from outpatient records. Patients were examined clinically; laboratory and radiological tests were performed annually during the follow-up. Postoperative diagnosis/treatment of diabetes and pancreatic enzyme replacement therapy were performed by the endocrinology and gastroenterology clinics, respectively.

### 2.1. Statistical analysis

Continuous data were expressed as mean ± standard deviation (SD). Categorical data were reported as frequencies. Association between dichotomous and continuous data was analyzed using point-biserial correlation test; in order to perform Pearson’s correlation test, either normality of the data should be checked, or nonparametric Spearman’s test should be preferred. Group differences with continuous data were analyzed using independent samples *t* test. Data were statistically analyzed using SPSS version 22.0 software (IBM Inc.; Armonk, NY, USA). Statistical significance was set at <0.05.

## 3. Results

Medical charts of patients diagnosed with insulinoma were retrospectively identified, and analyzed at the Azerbaijan Medical University. Twenty-one patients were included in the study. The majority (52.4%) of patients were male. The mean age was 44.2 ± 17.2 year-old and the mean time from symptoms onset to diagnosis was 14 ± 13.9 months (Table 1). Out of 21 patients, 17 (80%) had a history of at least one referral, while 13 (61%) patients had ≥ 3 referrals to various medical facilities due to complaints related to hypoglycemia. Neurology was the most commonly referred clinic. Whipple’s triad was present in all patients at the time of admission. The mean glucose, insulin, C-peptide levels at admission were 35.7 ± 9.5 mg/dL, 33.5 ± 21.9 µU/mL, and 3.74 ± 1.88 ng/mL, respectively. The most common symptoms during admission were syncope (76%), sweating (65%), and palpitation (41%).

**Table 1:** Demographic data of insulinoma patients.

Number of patients: n (%)	Male: 11 (52.4) Female: 10 (47.6)
Age (Mean ± SD)	Overall: 44.2 ± 17.2 years Female: 41.2 ± 20^a^ years Male: 46.9 ± 14.5^a^ years
Time from onset of symptoms to diagnosis (Mean ± SD)	Overall: 14 ±13.9 months Female: 13.8 ±11.5^b^ months Male: 15.09 ±16.6^b^ months
Biochemical tests to confirm insulinoma (Mean ± SD)	Insulin level: 33.5 ± 21.9 µU/mL C-peptid: 3.74 ± 1.88 ng/mL Glucose: 35.7 ± 9.5 mg/dL
Frequency of symptoms at admission (%)	Syncope (76) Sweating (65) Tachycardia (41) Tremor (35) Vertigo (29) Sleep disorder (24) Weight gain (24)

^a^ Group differences were not statistically significant (P = 0.46). _b_Group differences were not statistically significant (P = 0.83).

**Figure 1:**
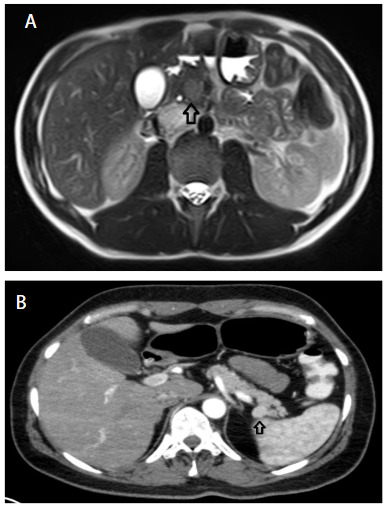
(A) MRI (T2A) images of the abdomen. Arrow shows the hyperintense insulinoma in the pancreatic head. (B) Arterial phase CT image of the abdomen. Arrow shows hypervascular, hyperdense mass lesion in the pancreatic tail.

**Figure 2:**
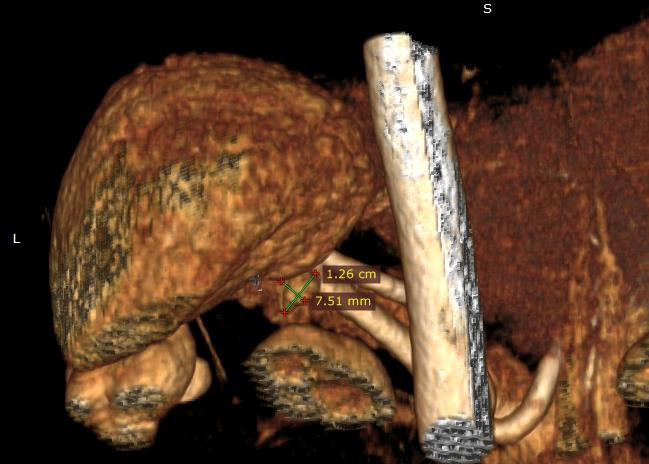
CT-angio image of insulinoma in the pancreatic tail (posterior view).Tumor size is shown.

US, CT, and MRI were the most frequently preferred imaging techniques used. In order to localize the tumor, In-111 octreotide scintigraphy was performed in two (9.5%) patients and ASVS was used in three (14.2%). The sensitivity of CT, MRI and US was 85% (12/14), 80% (12/15), and 55% (6/11), respectively (Figures 1 and 2).

Pancreatic head and tail were the most frequent location of the tumors that comprised eight (38%) and seven (33.3%) patients, respectively (Table 2). Macroscopic examination of the resected specimens showed that the mean tumor diameter was 1.5 ± 0.7 cm. There was no correlation between tumor size and parameters of preoperative glucose, C-peptide and insulin levels (P > 0.05). 

**Table 2:** Location of resected insulinomas and surgical procedures performed.

Location: n (%)	Head 9 (42.8) Tail 7 (33.3) Body 4 (19) Neck 1 (4.7)
Surgical procedures: n (%)	Distal pancreatectomy 8 (38) Enucleation 6 (28.7) Whipple procedure 4 (19) Total pancreatectomy 2 (9.5) Segmental pancreatectomy 1 (4.7)

**Figure 3:**
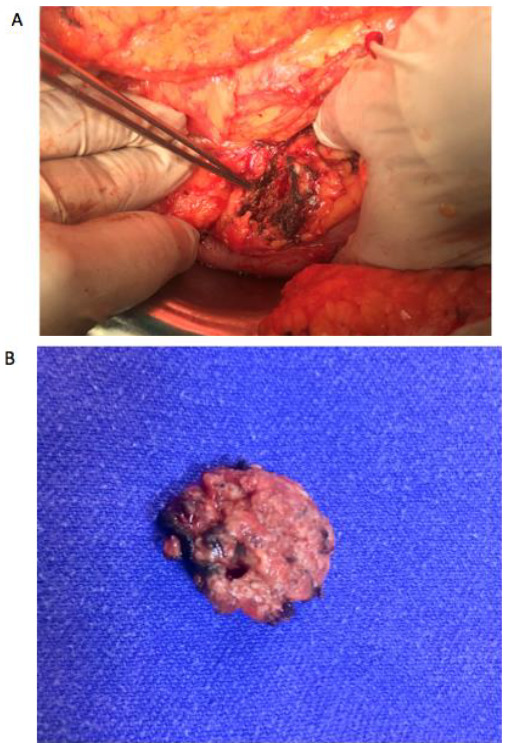
(A) Intraoperative view of pancreatic head after enucleation performed and hemostasis achieved. (B) Enucleated tumor.

**Figure 4:**
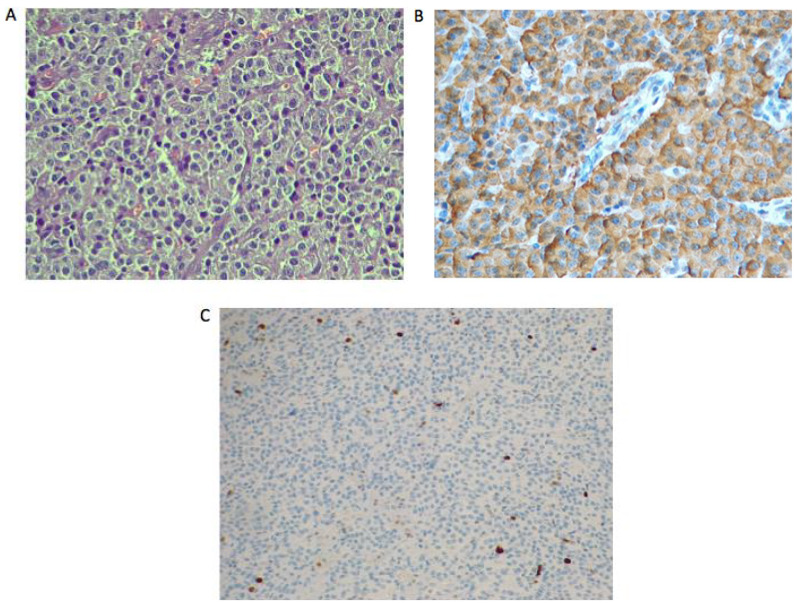
Histopathological examination of insulinoma. (A) Solid proliferation of monomorphic cells with large cytoplasm and round nuclei. Immunohistochemical analysis showed (B) chromogranin stained cells, (C) synaptophysin positive stained cells. The cells were also CD56 positive. Ki67 proliferation index was 4%–5% in tumor cells.

During the laparotomy, the operating surgeon suspected the presence of multiple pancreatic tumor in two (9.5%) patients, which was confirmed by intraoperative ultrasonography. The ultrasound also revealed that the tumors were in close relationship with the main pancreatic duct (< 2 mm). Thus, the decision was to perform a total pancreatectomy. Multiple endocrine neoplasia (MEN-1 syndrome) was diagnosed in one of these patients. Distal pancreatectomy was performed in eight cases (38%), enucleation in and six (28.7%), Whipple procedure in four (19%), total pancreatectomy in two (9.5%), and segmental pancreatectomy in one (4.7%) case (Table 2) (Figures 3 and 4). 

During the median 36-month follow-up period (range: 3–60 months), local tumor recurrence was found in three (14.2%) male patients (Table 3). Two (9.5%) patients presented with metastatic lymphadenopathy close to the resection margin, which was located by CT imaging. These two patients underwent radical lymphadenectomy and received adjuvant chemotherapy. A Whipple procedure was initially performed on one of these patients and a second tumor appeared close to the resection site. The patient underwent total pancreatectomy, lymphadenectomy, and adjuvant chemotherapy. None of these patients presented with hepatic metastases.

**Table 3:** Details of the cases with local recurrence.

Preoperative tumor location	Tumorsize (cm)	Surgicalprocedure	Pancreaticrecurrence	Lymph node metastasis
Tail*	0.8	Total pancreatectomy	-	+
Neck	1.6	Whipple procedure	+	+
Head	1.8	Whipple procedure	-	+

* Multiple tumors were found intraoperatively.

Although laparoscopic cancer surgery has been steadily increasing in Azerbaijan, only two (9.5%) insulinoma patients were operated laparoscopically. Enucleation of insulinoma from the pancreatic head was performed in one patient, while the other underwent a laparoscopic spleen-preserving distal pancreatectomy due to an insulinoma in the pancreatic tail. 

Complete resolution of hypoglycemic symptoms and clinical improvement was achieved in all patients postoperatively. Two total pancreatectomy and one distal pancreatectomy patients became diabetic (14.2%) in the early postoperative period. New onset malabsorption that required enzyme replacement therapy was also found in these three (14.2%) patients. There was no perioperative or postoperative mortality. The mean length of stay was 13.4 ± 8.2 days. The most common postoperative complication was ‘biochemical leak’ from the pancreas, which occurred in four (19%) patients. Two (9.5%) cases were due to enucleation and the other two (9.5%) occurred after Whipple procedures. These patients required prolonged use of intraabdominal drains. Surgical site infections were diagnosed in three (14.2%) patients, which was treated by partial removal of surgical sutures, frequent dressing changes and tertiary wound closure.

## 4. Discussion

Although insulinomas are a rare disease, they are the most commonly diagnosed type of functional PanNET. Most cases reported in the literature are either case reports, case series or reviews [10–17]. Despite the nonspecificity of its symptoms, typical severe fasting hypoglycemia in nondiabetics and presence of Whipple triad should suggest a possible diagnosis of insulinoma, even may present with postprandial severe hypoglycemia [18]. Failure to recognize these symptoms may lead to delays in diagnosis. Our data showed that the mean symptoms from onset to diagnosis was 14 months and 80% of patients had sought previous medical attention. 

Sporadic insulinomas have been reported to occur ‘anytime in life’, they seem to affect both genders equally, and the mean age at presentation is 47 years [19]. Similarly, we report a mean age of 44 years (range: 21–80). Overall, 90.5% of cases had solitary pancreatic tumors in our series and except for the case associated with MEN-1 syndrome, 95% of sporadic insulinomas had solitary tumors. Likewise, literature data shows that approximately 90% of insulinomas are sporadic, and tend to be solitary and treatable by limited resection [20]. Although literature shows that insulinomas may be located anywhere in the pancreas, the majority of tumors have been reported to be located in the body and tail and their sizes are smaller than 2 cm, whereas malignant tumors are usually over 3 cm in diameter [20–23]. Our report showed that 76.1% of insulinomas were located in the head and tail of pancreas and the mean tumor size was 1.5 cm. Interestingly, the recurrent tumor size in our study was 1.4 cm. 

Imaging techniques, including intraoperative ultrasound, are critical for planning the most appropriate surgical procedure. Dynamic CT has replaced conventional CT technique due to its ability to detect smaller tumors, which increase the sensitivity of CT to 80%. CT also may exclude the presence of metastases [6]. Combining biphasic helical CT and endoscopic US has been reported to demonstrate 100% sensitivity in localizing insulinomas [24]. Literature data also show that MRI is important for the detection of small diameter insulinomas that were not detected by transabdominal US or CT [6,25,26]. Octreotide scintigraphy is of limited use in sporadic insulinomas, its sensitivity is approximately 40%–50%.6 Although it is an invasive procedure with potential for complications, ASVS is especially important when other imaging techniques fail to identify insulinomas [6]. In our series, the sensitivity for localizing pancreatic tumors of CT, MRI and US was 85%, 80%, and 55%, respectively. Our series showed that the sensitivity of ASVS was 100% when other imaging techniques failed to identify insulinomas. Tactile information revealed that by palpating the pancreatic gland might be helpful for tumor localization during surgery. Moreover, intraoperative Doppler US was particularly helpful and accurate for detecting multiple insulinomas in our series. Doppler US also reveals the hypervascularity of the tumor, which is suggestive for insulinomas [12].

Crippa et al. reported [24] on the surgical management of insulinomas bring perspective to our understanding for tumor recurrences. They reported that positive tumor margins were found in 4.5% of their enucleation cases. Moreover, no lymph nodes were excised in 87%, 75%, and 70% of enucleation, middle pancreatectomy and spleen preserving distal pancreatectomy operations, respectively. Likewise, in the follow-up period, two patients (9.5%) presented with metastatic lymph nodes after the initial resections, which may be due to the low lymph node yield of routinely performed insulinoma surgeries. We also advocate for Crippa et al. findings that suggest peripancreatic lymph node sampling during routine insulinoma surgeries in order to decrease local recurrences. We also speculate that this may be generalized to other PanNET surgeries. One patient presented with a second tumor close to the resection site, and it was likely a second primary tumor that was found at 36 months after the initial surgery.

There were no mortalities in our series. Pancreatic fistula has been reported to constitute the majority of complications (3%–60%) after pancreatic resections [9]. In our series, 19% of patients presented with low grade, ‘biochemical leaks’, that required prolonged presence of intraabdominal suction drains. Intraoperative US and endoscopic US may help determine the relationship between insulinoma and pancreatic duct before proceeding with enucleation. 

This study is not without limitations. Prospective study design for insulinomas is not practical as they are not a frequent disease. Retrospective design inevitably led to data constraints that led to unanswered questions. Tumor stage and grade data were not uniformly present in patient records. We were also unable to find any admissions that presented with metastatic insulinomas. This is the result that this study is a single center report and a comprehensive database does not exist yet. As the population included in the study is small, statistical analyses were not suitable in many instances and results need cautious interpretation. One interesting example is the lack of any association between tumor size, plasma C-peptide, and insulin levels. Moreover, as neither 5- nor 10-year follow-up of the patients have been finished, we are unable to present relevant survival data. 

In conclusion, surgery is still the treatment of choice for insulinomas. Unfortunately, delays in diagnosis still occur and treating physicians should suspect insulinoma diagnosis when typical symptoms are encountered. Laparoscopic enucleation and pancreatic resection will likely dominate the future surgical treatments of this disease. However, implementation of laparoscopic surgery seems to require the availability of endoscopic US in order to elucidate multiple, undetected tumors and their relationship with the main pancreatic duct.
